# Metabolomics-Based Analysis of Quality Differences in *Dendrobium officinale* Leaves Under Different Flower Treatments

**DOI:** 10.3390/metabo16090643

**Published:** 2026-09-02

**Authors:** Xiaoxu Ren, Wu Ying, Lihua Chen, Bingbin Zheng, Shouzeng Xu, Le Zhang, Xianbo Wang, Luxia Chen, Bowei He, Songlin Ruan

**Affiliations:** 1 Institute of Crop Science, Hangzhou Academy of Agricultural Sciences, Hangzhou 310024, China; renxxhz@163.com (X.R.); hzyingwu@163.com (W.Y.); 2 Hangzhou Forest and Wetland Science Research Institute, Hangzhou 310005, China; lhchen2026@163.com; 3 Zhejiang Yaoyuan Agricultural Development Co., Ltd., Hangzhou 311516, China; 13335711255@163.com; 4 Tonglu County Agricultural Technology Extension Center, Hangzhou 311599, China; 13325916390@163.com (S.X.); 15726829622@163.com (L.C.); 5 Experimental Center, Hangzhou Academy of Agricultural Sciences, Hangzhou 310024, China; sky6609@163.com (L.Z.); chayesuo2008@163.com (X.W.); 6 Zhejiang Agricultural Technology Extension Center, Hangzhou 310020, China

**Keywords:** *Dendrobium officinale*, flower, leaf quality, free amino acids, source–sink relationship, carbon–nitrogen partitioning, secondary metabolites

## Abstract

**Background/Objectives**: To elucidate how flower retention regulates leaf quality formation in *Dendrobium officinale* and to provide a theoretical basis for high-quality leaf cultivation and high-value utilization of byproducts, *D. officinale* leaves were subjected to two treatments: flower retention (FR) and flower removal (FT). **Methods**: Quantitative analysis revealed significant differences in quality-related compounds between the two treatments. Specifically, the polysaccharide content in the FR group (8.72%) was significantly lower than that in the FT group (9.37%). Conversely, the FT group exhibited a significantly higher total flavonoid content (1.74%) compared to the FR group (1.41%). Additionally, the total phenol content in the FR group (0.90%) was significantly higher than that in the FT group (0.69%) (*n* = 3). **Results**: Compared with the FR group, the FT group exhibited significantly higher levels of total flavonoids, polysaccharides, tyrosine (Tyr), hydroxylysine (Hylys), lysine (Lys), histidine (His), and arginine (Arg). A total of 1999 (39.5%) of the 5062 annotated metabolites were accumulated significantly differently. The FT group was mainly characterized by upregulated secondary metabolites, including flavonoids. Kyoto Encyclopedia of Genes and Genomes (KEGG) enrichment analysis indicated that nucleotide metabolism, purine metabolism, aminoacyl-tRNA biosynthesis, and flavonoid biosynthesis were the core differential pathways. Furthermore, the differential metabolites exhibited a metabolic trade-off of “upregulated secondary metabolism and downregulated primary metabolism”. **Conclusions**: Our findings indicated that flower removal can significantly increase the active flavonoid components and amino acids in *D. officinale* leaves and optimize the quality of this medicinal leaf by regulating the source–sink relationship, carbon–nitrogen partitioning, and key metabolic pathways.

## 1. Introduction

*Dendrobium officinale* Kimura et Migo, a perennial epiphytic herb belonging to the *Orchidaceae* family, is a well-known traditional Chinese medicinal material that has the effects of nourishing yin and clearing heat, benefiting the stomach, and enhance biosynthesis of body fluids [1]. *D. officinale* has been widely used in medicinal and edible foods, pharmaceuticals, cosmetics, and other fields and has a continuously growing market demand [2]. With the rapid development of the *D. officinale* industry in recent years, its leaves, as a byproduct, have attracted increasing attention in addition to its traditionally used medicinal stem. In 2017, the National Health Commission of China approved *D. officinale* leaf as a medicinal and edible plant resource. *D. officinale* leaves are abundant in polysaccharides, flavonoids, phenolics, free amino acids, and other bioactive components with high development and utilization values, thereby representing an important direction for expanding the utilization of *Dendrobium* resources [3,4,5]. However, the quality of *D. officinale* leaves is affected by several factors such as variety, cultivation mode, growth environment, and management measures. Among them, flower retention or removal, as a key cultivation regulation measure, may affect nutrient distribution, source–sink relationship, and metabolic flux in plants, thereby influencing the accumulation and distribution of bioactive components in leaves [6,7,8,9,10,11]. Nevertheless, systematic studies on the effect of this measure on the leaf quality of *D. officinale* remain limited.

Flowers act as important sink organs during plant growth and development. They compete with source organs such as leaves for photosynthates, mineral nutrients, and other metabolites, thus regulating nutrient allocation and physiological metabolism [6]. Studies have shown that flower removal can reduce nutrient distribution to reproductive organs, promote the accumulation of photosynthetic products, and increase the synthesis of secondary metabolites in vegetative organs. The flowering process bears an intimate relationship with the post-anthesis vegetative growth of *Dendrobium officinale*; profuse floral development results in dramatic consumption of reserved nutrients required for vegetative growth [12]. Flower thinning or removal can significantly increase the contents of soluble sugars, vitamins, and secondary metabolites in the leaves and improve the quality of fruits or vegetative organs [13]. In *Dendrobium* species, flower development consumes a large amount of carbohydrates and nitrogen nutrients. As the main source organ, leaves are the major site for photosynthate synthesis and nitrogen assimilation, and the presence of flowers may significantly affect carbon–nitrogen metabolism and the accumulation of bioactive components in leaves [14]. Furthermore, several studies have confirmed that flower removal during the flowering stage of *D. officinale* significantly increases the contents of polysaccharides in stems and improves their medicinal quality [15,16]. However, the effects of flower retention on the accumulation of free amino acids, polysaccharides, flavonoids, phenols, and other active components in leaves, as well as the underlying regulatory mechanism, remain unclear, restricting the optimization and popularization of high-quality cultivation techniques for *D. officinale* leaves.

As a high-throughput and unbiased method of metabolic analysis, nontargeted metabolomics can systematically detect changes in all small-molecule metabolites in biological samples and comprehensively reveal the regulatory mechanisms underlying plant metabolic networks under different treatments. This analytical technique has been widely used in cultivation regulation, exploration of quality formation mechanisms, and metabolic pathway analysis of medicinal plants [17,18]. It can simultaneously detect changes in the contents of hundreds of metabolites. When combined with multivariate statistical analysis, untargeted metabolomics can be used to screen differential metabolites and analyze their associated pathways, thereby providing robust technical support for elucidating the regulatory mechanisms by which cultivation measures shape the quality of medicinal plants. In recent years, metabolomics has been used to analyze metabolic differences in different tissues, growth stages, and cultivation modes of *D. officinale*, including metabolomic comparison of stems from different origins and metabolic profiling of *D. officinale* at different growth years, thereby providing an important reference for quality evaluation and regulatory mechanism research [19,20]. However, to the best of our knowledge, metabolomics-based analysis of the effects of flower retention on the leaf metabolome of *D. officinale* has not been reported, and its regulatory mechanism remains unclear.

In this study, *D. officinale* leaves were assigned to two cultivation treatments: flower retention (FR) and flower removal (FT). The alcohol-soluble extract and the contents of free amino acids, polysaccharides, total flavonoids, and total phenols were systematically determined. Moreover, in combination with nontargeted metabolomics, the metabolic differences in leaves under different flower conditions were analyzed, key differential metabolites were screened, and related metabolic pathways were annotated. This study clarifies the regulatory mechanisms by which flower retention influences leaf quality formation in *D. officinale*, provides a theoretical basis for optimizing high-quality cultivation techniques, and supports the high-value utilization of *D. officinale* leaf byproducts.

## 2. Materials and Methods

### 2.1. Sample Collection

*D. officinale* leaf samples were collected from the cultivation base of Zhejiang Yaoyuan Agricultural Development Co., Ltd. (Hangzhou, China). Three-year-old *D. officinale* plants were cultivated in a greenhouse under natural light with regular irrigation. The plants were arranged in a completely randomized design. Flower removal treatments were conducted during the flowering period in May, and the 3rd to 5th fully expanded current-year leaves were harvested in August, three months after the treatment. For the analysis, leaf samples from each treatment group were collected, with approximately 200 g of fresh weight for each biological replicate (*n* = 5). In August 2025, the leaves were sampled from plants subjected to two treatments in the following groups: FR and FT. A portion of the fresh samples was stored at −80 °C for free amino acid analysis. The remaining fresh samples were dried in an oven at 80 °C, ground into a powder, and sieved through a 60-mesh sieve to obtain test samples. The contents of alcohol-soluble extract, total phenols, total flavonoids, polysaccharides, and amino acids were measured using three biological replicates. For metabolomic profiling, five biological replicates were prepared for each group.

### 2.2. Determination of the Bioactive Components in D. officinale Leaves

Total flavonoids were determined by ultraviolet spectrophotometry at 505 nm using the NaNO_2_-Al(NO_3_)_3_-NaOH color development method with rutin as the standard reference [21]. Total phenols were quantified at 733 nm using gallic acid as the standard [22], and polysaccharides were quantified at 490 nm using anhydrous glucose as the standard [23]. Free amino acids were analyzed according to the national standard method GB/T 30987-2020 [24].

### 2.3. Metabolite Extraction and Liquid Chromatography (LC)-Mass Spectrometry (MS)/MS

A 100-mg sample of the dried leaf powder was ground at room temperature, transferred to an Eppendorf tube, and mixed with 500 μL of 80% aqueous methanol. After vortexing and incubation in an ice bath for 5 min, the mixture was centrifuged at 15,000× *g* and 4 °C for 20 min. An aliquot of the supernatant was diluted with mass-grade water to a methanol concentration of 53% and recentrifuged at 15,000× *g* for 20 min at 4 °C. The supernatant was collected for LC-MS. The quality control (QC) samples were prepared by pooling equal volumes of all experimental sample extracts. They were injected at the beginning, middle, and end of the LC-MS analytical sequence, with one QC injection inserted after every 20 experimental samples. During data preprocessing, the coefficient of variation (CV) was calculated for all metabolites across the QC samples, and those exhibiting a CV exceeding 30% were excluded from further analysis.

HPLC-MS/MS analyses were performed using a Vanquish UHPLC system (Thermo Fisher, Dreieich, Germany) coupled with an Orbitrap Q ExactiveTM HF massspectrometer or Orbitrap Q ExactiveTMHF-X mass spectrometer (Thermo Fisher, Dreieich, Germany) in Novogene Co., Ltd. (Beijing, China). Samples were injected onto a Hypersil Goldcolumn (100 mm× 2.1 mm, 1.9 μm) using a 12-min linear gradient at a flowrate of 0.2 mL/min. The eluents for the positive and negative polarity modes were eluent A (0.1% FA in Water) and eluent B (Methanol). The solvent gradient was set as follows: 2% B, 1.5 min; 2–85% B, 3 min; 85–100% B, 10 min; 100–2% B, 10.1 min; 2% B, 12 min. Q Exactive^TM^ HF mass spectrometer was operated in positive/negative polarity mode with spray voltage of 3.5 kV, capillary temperature of 320 °C, sheathgas flow rate of 35 psi and aux gas flow rate of 10 L/min, S-lens RF level of 60, Auxgas heater temperature of 350 °C. Full scan MS spectra were acquired at a resolution of 60,000 FWHM (at *m*/*z* 200) with an AGC target of 3 × 10^6^ and a maximum injection time of 100 ms. Data-dependent MS/MS scans were acquired at a resolution of 15,000 FWHM with an AGC target of 2 × 10^5^ and a maximum injection time of 25 ms. The isolation window was set to 1.5 m/z. Stepped normalized collision energy (NCE) of 20, 40, and 60 eV was applied for fragmentation. Other DDA parameters included a dynamic exclusion time of 10 s, apex trigger of 2–12 s, and isotopic exclusion enabled. The instrument was calibrated regularly using the standard calibration solution to ensure mass accuracy.

### 2.4. Data Processing and Metabolite Identification

All data were expressed as mean ± standard deviation (SD) and the significance was analyzed by Student’s *t*-test using IBM SPSS Statistics 26. Raw data were converted to mzXML format using ProteoWizard (https://proteowizard.sourceforge.io/). Peak extraction, alignment, and quantification were performed using XCMS. Metabolites were annotated by comparison with the NovoMetDB database (Novogene) with a mass tolerance of 10 ppm.

These metabolites were annotated using the KEGG database (https://www.genome.jp/kegg/pathway.html) (accessed on 27 August 2026), HMDB database (https://hmdb.ca/ metabolites) (accessed on 27 August 2026) and LIPIDMaps database (http://www.lipidmaps.org/) (accessed on 27 August 2026). Principal components analysis (PCA) and Partial least squares discriminant analysis (PLS-DA) were performed at metaX.We applied univariate analysis (*t*-test) to calculate the statistical significance (*p*-value). FC refers to the fold change, which is the ratio of the mean of all biological repeated quantitative values of each metabolite in the comparison group. The differential metabolites were screened using VIP > 1, |log_2_FC| > 1, and BH-adjusted *p* value (FDR) < 0.05. For clustering heat maps, the data were normalized using z-scores of the intensity areas of differential metabolites and were ploted by Pheatmap package in R language. The correlation between differential metabolites were analyzed by cor () in Rlanguage (method = pearson). Statistically significant of correlation between differential metabolites were calculated by cor.mtest() in R language. *p*-value < 0.05 was considered as statistically significant and correlation plots were ploted by corrplot package in R language. The functions of these metabolites and metabolic pathways were studied using the KEGG database. The metabolic pathways enrichment of differential metabolites was performed, when ratio were satisfied by x/n > y/N, metabolic pathway were considered asenrichment, when *p*-value of metabolic pathway < 0.05, metabolic pathway were considered as statistically significant enrichment.

## 3. Results

### 3.1. Effects of Different Treatments on the Quality Indices of D. officinale Leaves

**Figure 1:**
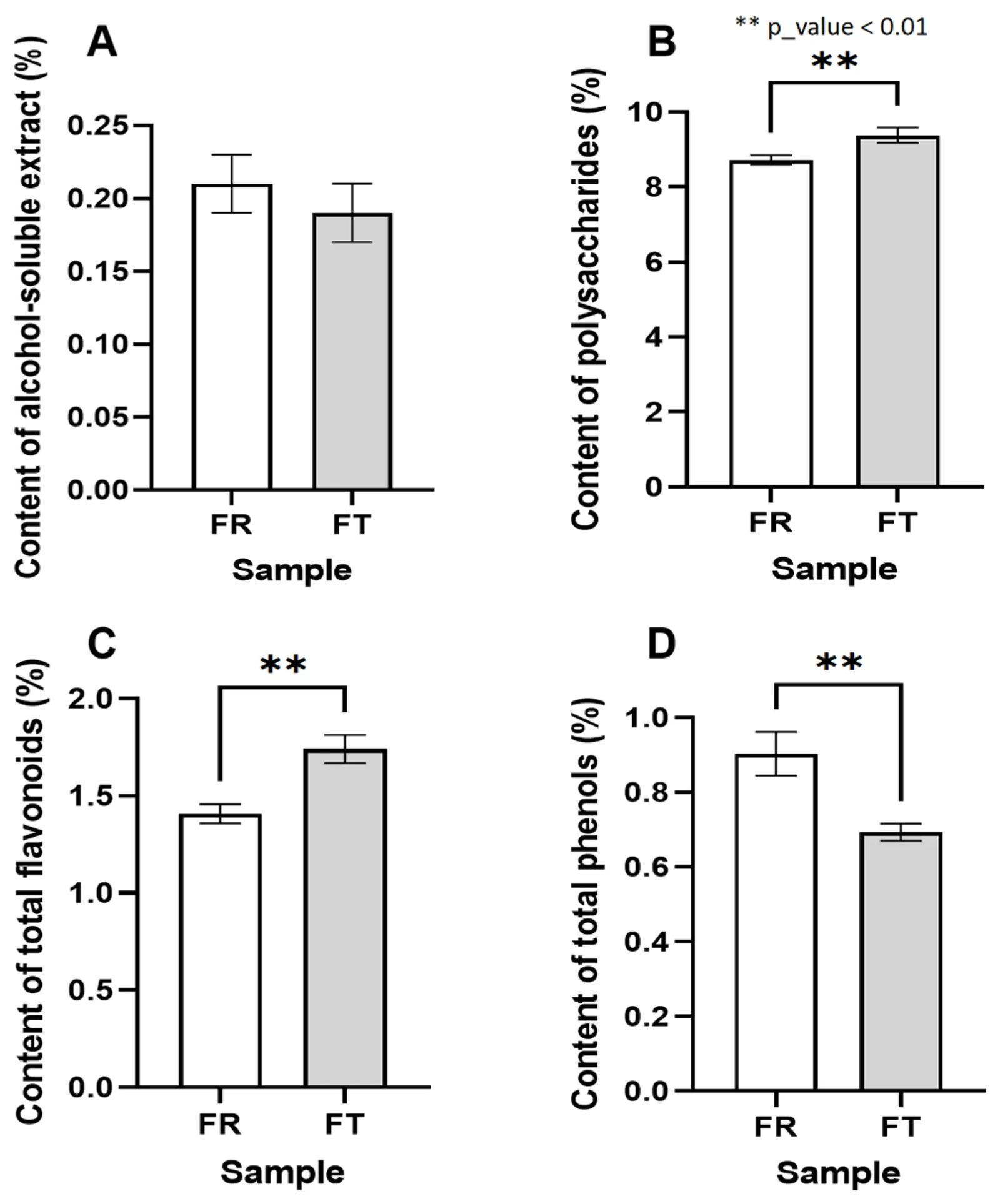
(**A**) The contents of alcohol-soluble extract in *Dendrobium officinale* leaves under different treatments. (**B**) The contents of polysaccharides in *Dendrobium officinale* leaves under different treatments. (**C**) The content of total flavonoids in *Dendrobium officinale* leaves under different treatments. (**D**) The content of total phenols in *Dendrobium officinale* leaves under different treatments. ** indicates significant difference at *p* < 0.01 level. Error bars represent s. d. (*n* = 3).

As shown in Figure 1, flower retention significantly affected the contents of polysaccharides, total flavonoids, and total phenols in the leaves (*p* < 0.01) but had no significant effect on the alcohol-soluble extract. Polysaccharide content in the FR group (8.72%) was significantly lower than that in the FT group (9.37%), indicating that flower removal favored the accumulation of polysaccharides. Total flavonoid content in the FT group (1.74%) was significantly higher than that in the FR group (1.41%), suggesting that flower removal promoted flavonoid biosynthesis and accumulation. Total phenol content in the FR group (0.90%) was significantly higher than that in the FT group (0.69%) (*p* < 0.01), indicating that flower retention facilitated the accumulation of phenols.

### 3.2. Composition and Content of Free Amino Acids

**Figure 2:**
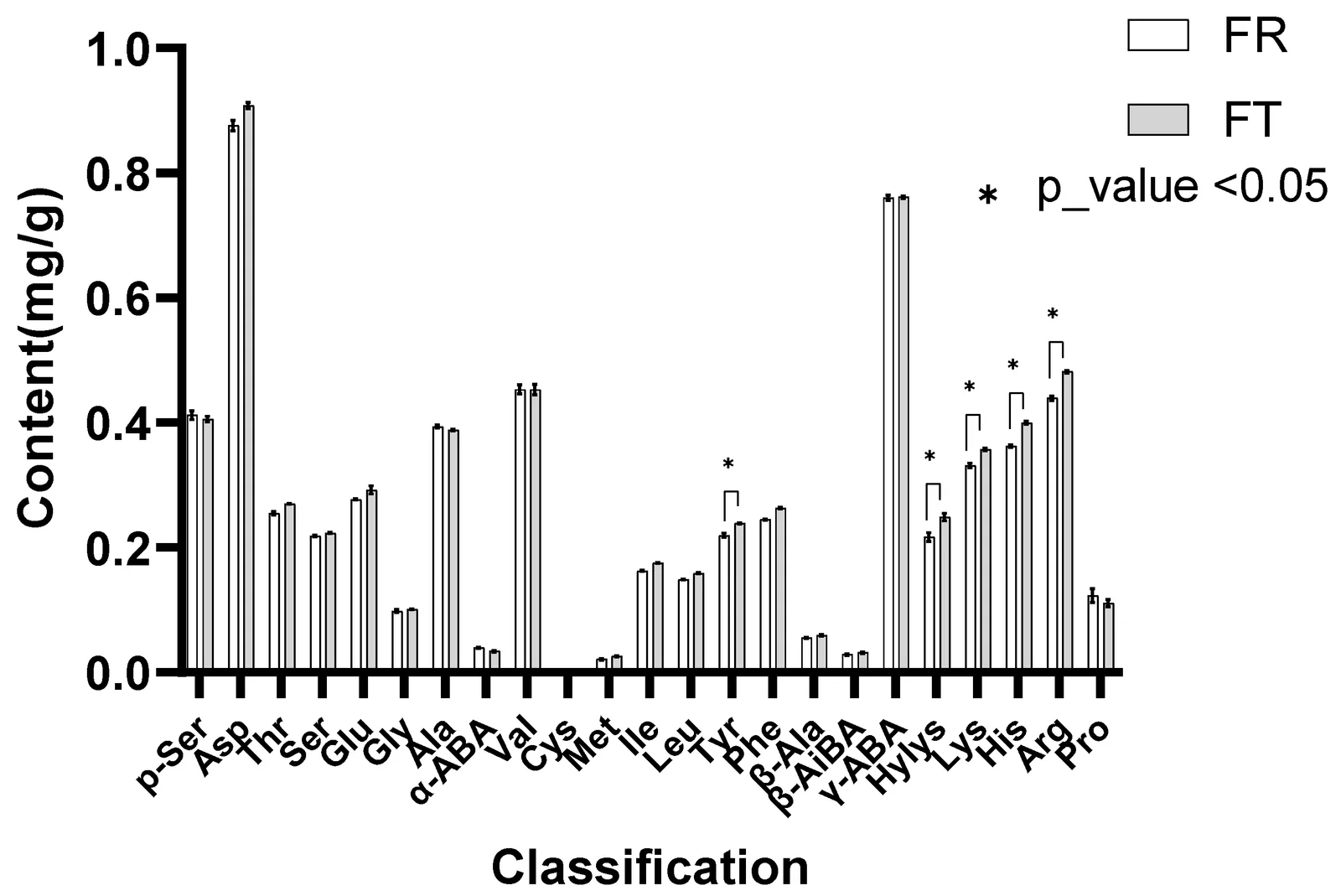
Composition and content of free amino acids in leaves of *Dendrobium officinale* under different treatments. * indicates significant difference at *p* < 0.05 level. Error bars represent s. d. (*n* = 3).

As shown in Figure 2, a total of 23 free amino acids were identified. Cysteine (Cys) was below the limit of detection. Aspartic acid (Asp) and *γ*-Aminobutyric acid (*γ*-ABA) exhibited relatively higher contents than other amino acids in both groups, with slightly elevated levels in the FT group without statistically significant differences. The contents of Tyrosine (Tyr), 5-Hydroxy-L-lysine (Hylys), Lysine (Lys), Histidine (His) and Arginine (Arg) were significantly increased in the FT group. Several amino acids including Phosphoserine (p-Ser), *γ*-ABA and Proline (Pro) showed reduced abundance in the FT group, yet no significant intergroup difference was detected. Methionine (Met), *β*-Alanine (*β*-Ala) and *β*-Aminoisobutyric acid (*β*-AIBA) maintained extremely low contents in both groups, and no significant variation was observed between the two treatments.

### 3.3. Metabolomic Analysis of D. officinale Leaves Subjected to Different Treatments

#### 3.3.1. Global Metabolic Characteristics and KEGG Functional Enrichment

**Figure 3:**
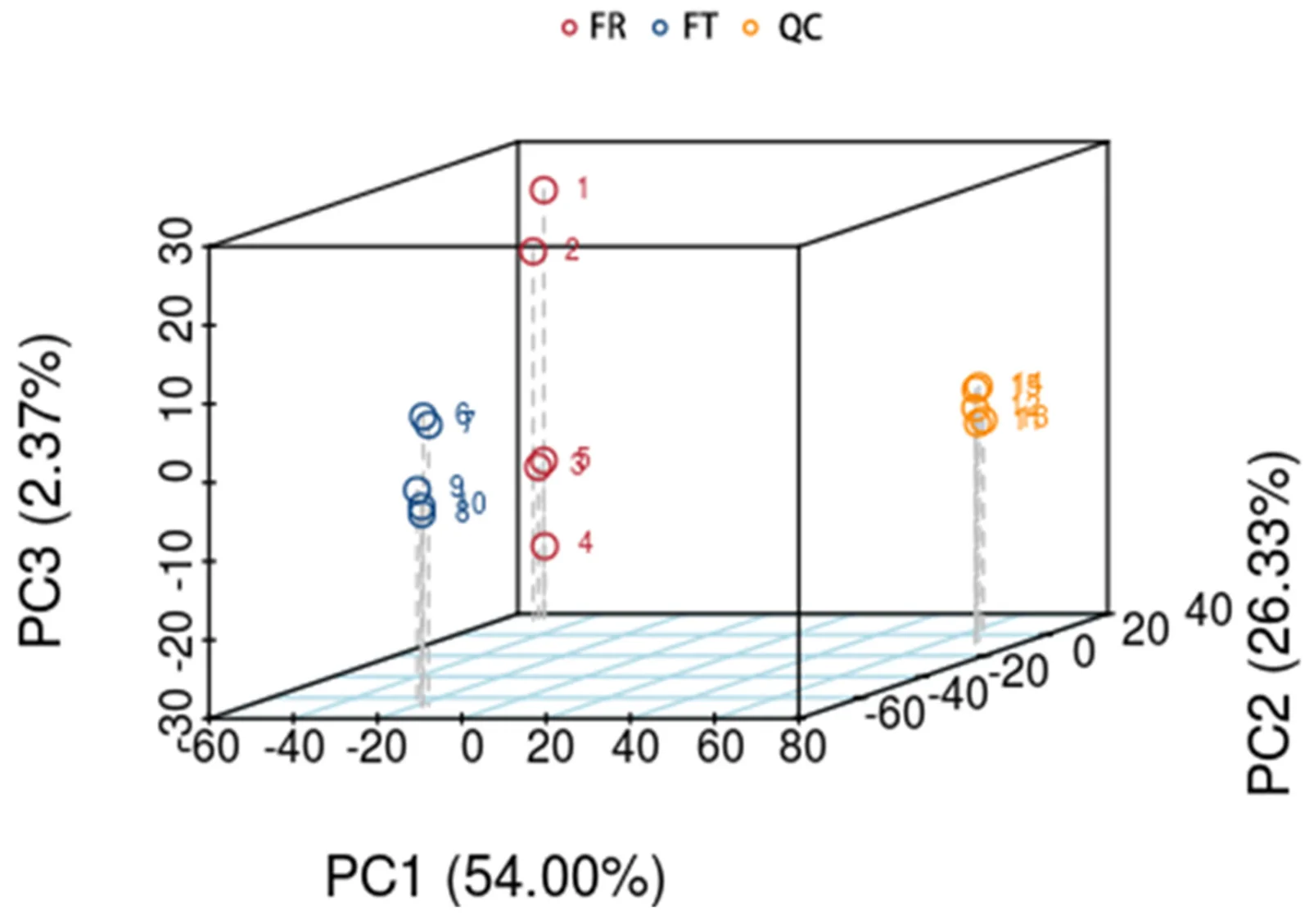
Principal component analysis (PCA) plot of samples from different treatment groups.

**Figure 4:**
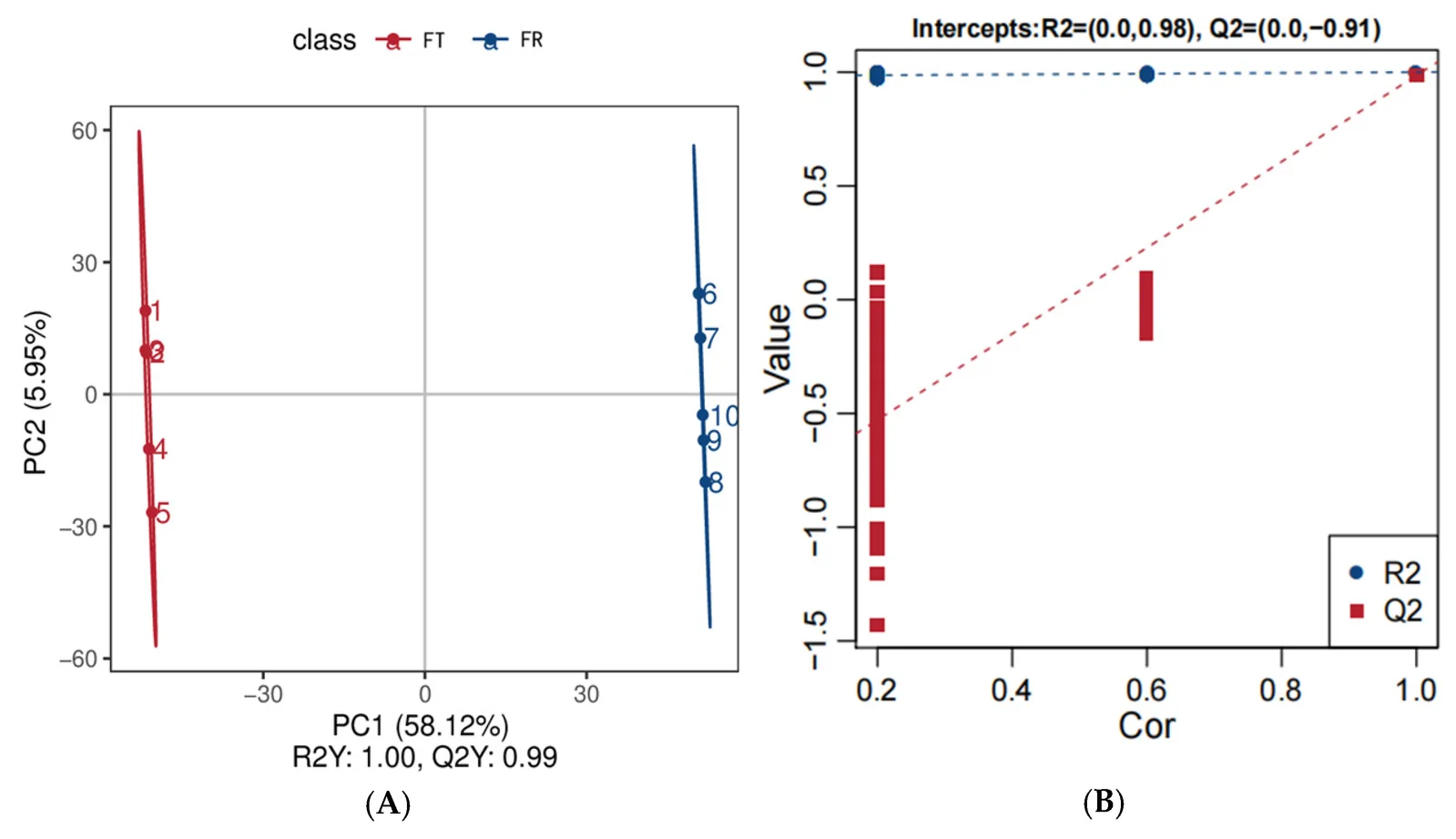

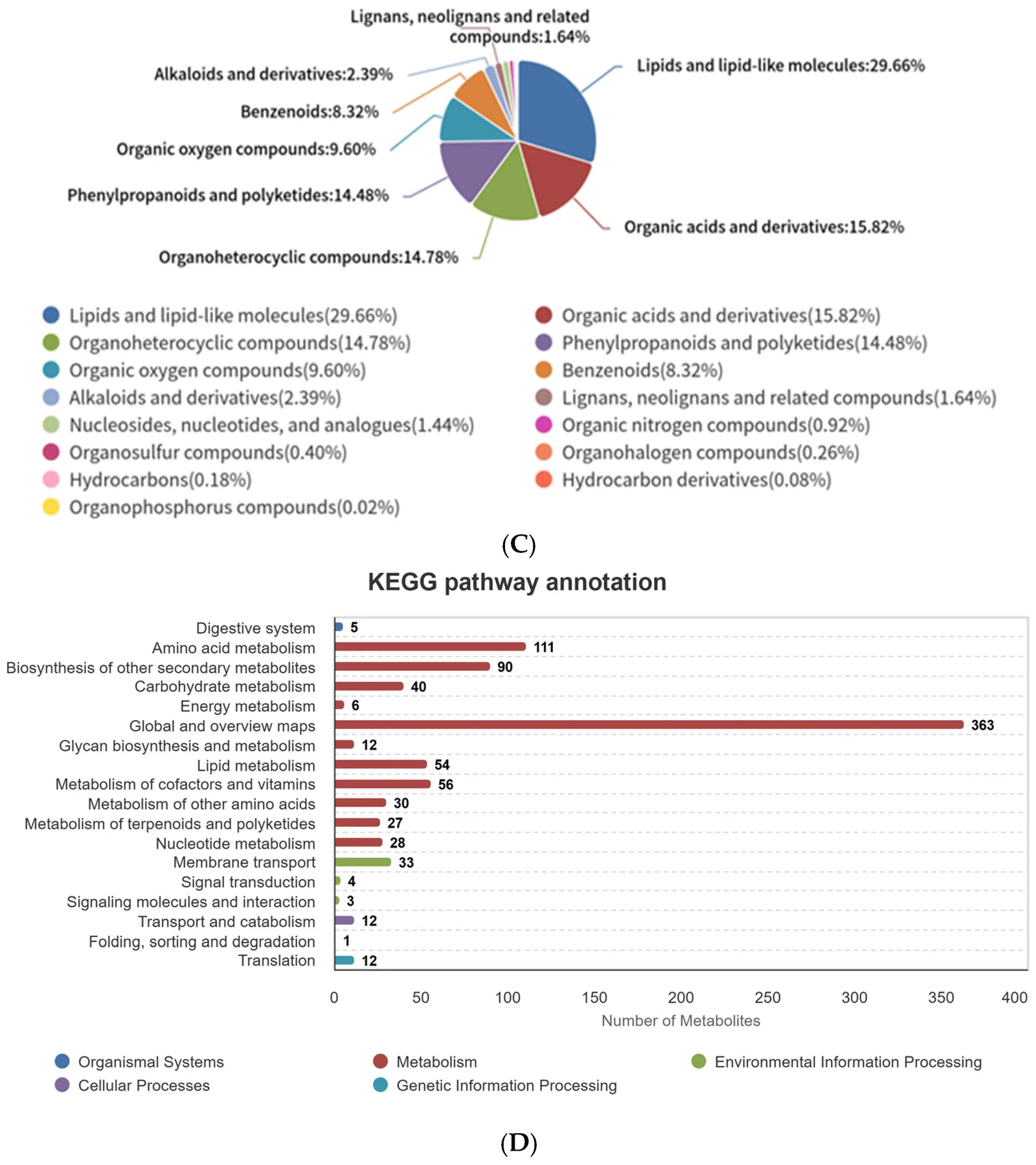
(**A**) OPLS-DA score plot of samples from different treatment groups. (**B**) OPLS-DA valid plot. (**C**) Chemical classification of total annotated metabolites. (**D**) KEGG pathway annotation of total metabolites.

PCA was used to reveal the overall metabolic differences. The first three principal components explained 54.00%, 26.33%, and 2.37% of the total variations, respectively, with a cumulative contribution rate of 82.70%, which well represented the overall metabolic characteristics (Figure 3). The 3D PCA score plot showed a clear separation between the FR and FT groups with tight clustering of biological replicates, indicating that flower removal could significantly reshape leaf metabolism. The data suggested good repeatability for subsequent differential metabolite screening and functional analysis. As shown in the OPLS-DA score plot (Figure 4A,B), a distinct separation was observed between the two groups. The model exhibited excellent explanatory and predictive capabilities (R^2^Y = 1.00, Q^2^ = 0.99). Furthermore, permutation testing confirmed that these parameters were significantly higher than those of the random distribution, demonstrating the robustness of the model and ruling out overfitting.

As seen in Figure 4C, classification of all annotated metabolites revealed lipids and lipid-like molecules to account for the highest proportion (29.66%), followed by organic acids and derivatives (15.82%), organic heterocyclic compounds (14.78%), and phenylpropanoids and polyketides (14.48%). These four classes accounted for over 74% of the total metabolites, indicating the dominance of lipid metabolism, organic acid metabolism, and secondary metabolism in leaves. The phenylpropanoid pathway, which encompasses flavonoids and phenolics, is the most central metabolic class involved in the differential variations of this study.

KEGG pathway annotation revealed that most metabolites were assigned to the Metabolism category. Global and overview maps (363), amino acid metabolism (111), and biosynthesis of other secondary metabolites (90) were the top three annotated terms, indicating that leaf metabolism focused on basic substance metabolism and secondary metabolic pathways, including flavonoids and phenylpropanoids (Figure 4D).

#### 3.3.2. Analysis of Differential Metabolites

A total of 5062 metabolite features were annotated, among which 1999 (39.5%) were significantly differential metabolites under the threshold *p* < 0.05, indicating a strong treatment-induced metabolic perturbation. Among them, 1337 metabolites were downregulated and 662 metabolites were upregulated, revealing an overall downregulation tendency.

**Figure 5:**
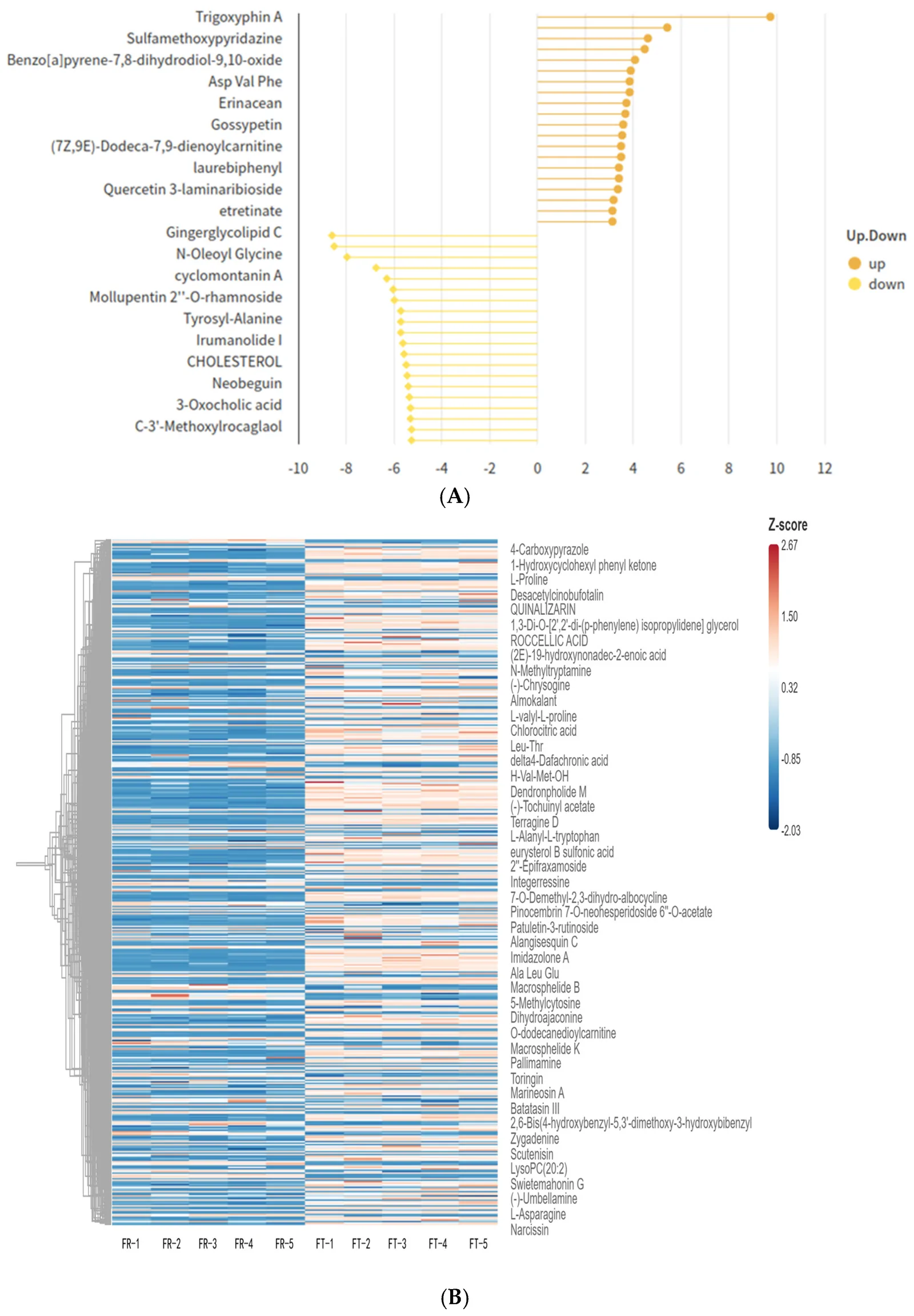

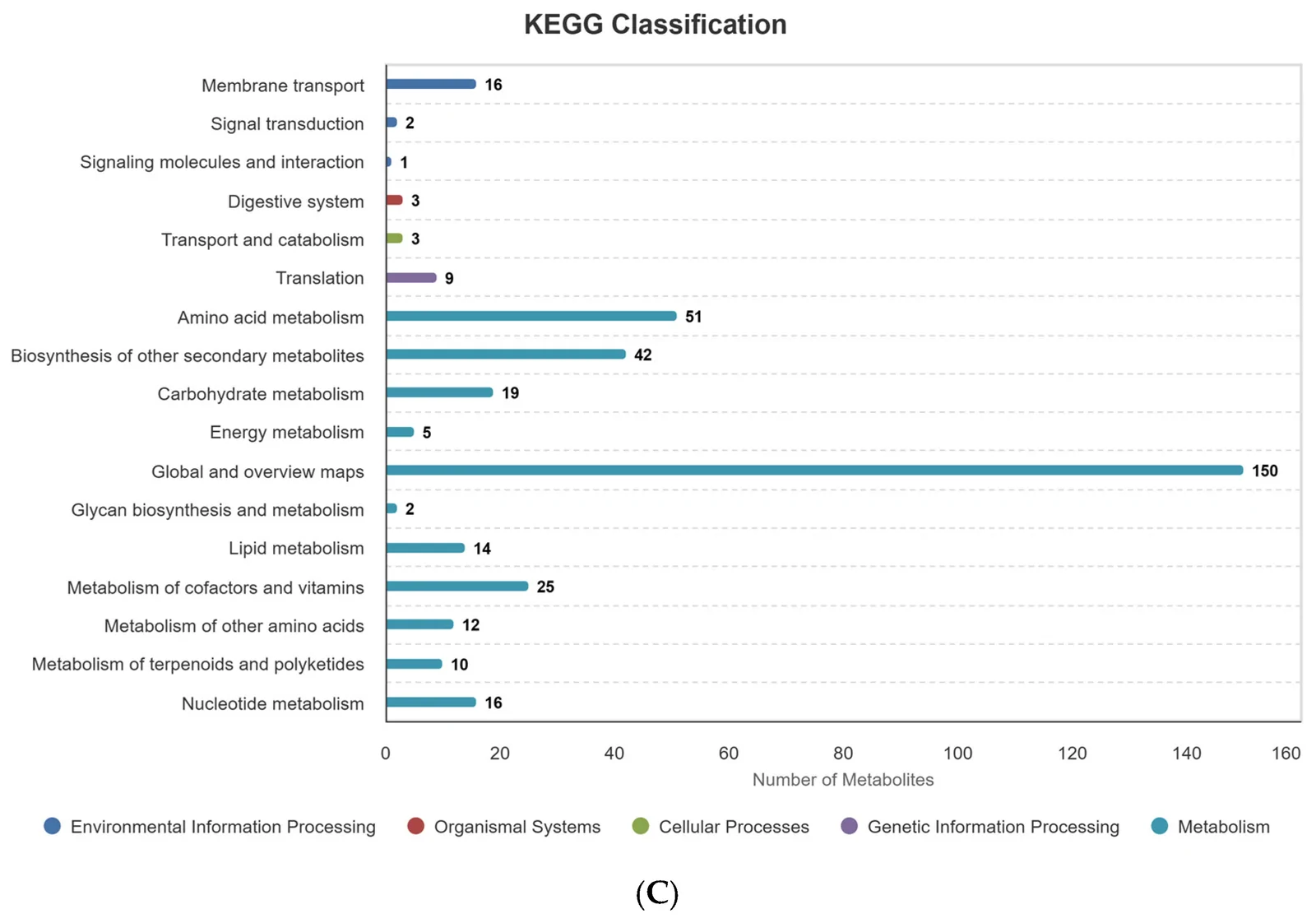
(**A**) Matchstick plot of differential metabolites (**B**) Cluster analysis of differential metabolites (**C**) KEGG annotation of differential metabolites.

Representative metabolites with the most significant FCs were screened (Figure 5A). gossypetin exhibited the highest upregulation level in the FT group. Multiple flavonoids, including herbacetin 8-acetate, bis-(4-hydroxybenzyl) sulfide, jasmalolactone B, morin, and 3′-methoxyflavonol, were all markedly upregulated. The upregulated metabolites were predominantly polyphenols belonging to flavones and flavonols, which was consistent with the results of our previous experiments. These plant-specific secondary metabolites are commonly associated with antioxidant and stress resistance pathways.

A pheatmap of differentially accumulated metabolites was constructed after row-wise Z-score normalization to visualize the expression patterns across all samples. The horizontal axis represented 5 biological replicates of the FR group and 5 replicates of the FT group, while the vertical axis indicated distinct metabolites with hierarchical clustering on the left (Figure 5B). Obvious separation was observed between the FR and FT groups. The dendrogram divided all metabolites into two major clusters: the upper cluster contained flavonoids and sulfur-containing compounds specifically enriched in the FT group, while the lower cluster consisted of phenolic compounds that accumulated abundantly in the FR group and were downregulated under FT group. These findings were mutually validated by the results of the fold-change bar plot. Based on the detected features, compounds including 4-carboxypyrazole, sapienic acid, bis-(4-hydroxybenzyl) sulfide, and 7,8,3′,4′-tetrahydroxyflavone were consistent with the upregulated metabolites (flavonoids and sulfides) described above, which were significantly accumulated under FT group. In contrast, substances such as adenosine, (-)-umbellamine, diathin E and tracheloside exhibited markedly decreased contents after FT group.

#### 3.3.3. KEGG Functional Annotation and Pathway Enrichment of Differential Metabolites

KEGG annotation of the differential metabolites revealed predominant enrichment in metabolism. Global and overview maps reveal the most annotated metabolites (150), followed by amino acid metabolism (51) and biosynthesis of other secondary metabolites (42), indicating differential metabolism to occur mainly in basic substance metabolism and secondary metabolite synthesis (Figure 5C). Furthermore, several differential metabolites were annotated to the metabolism of cofactors and vitamins, nucleotide metabolism, and carbohydrate metabolism, reflecting the significant regulation of energy metabolism, cofactor metabolism, and nucleic acid metabolism.

**Figure 6:**
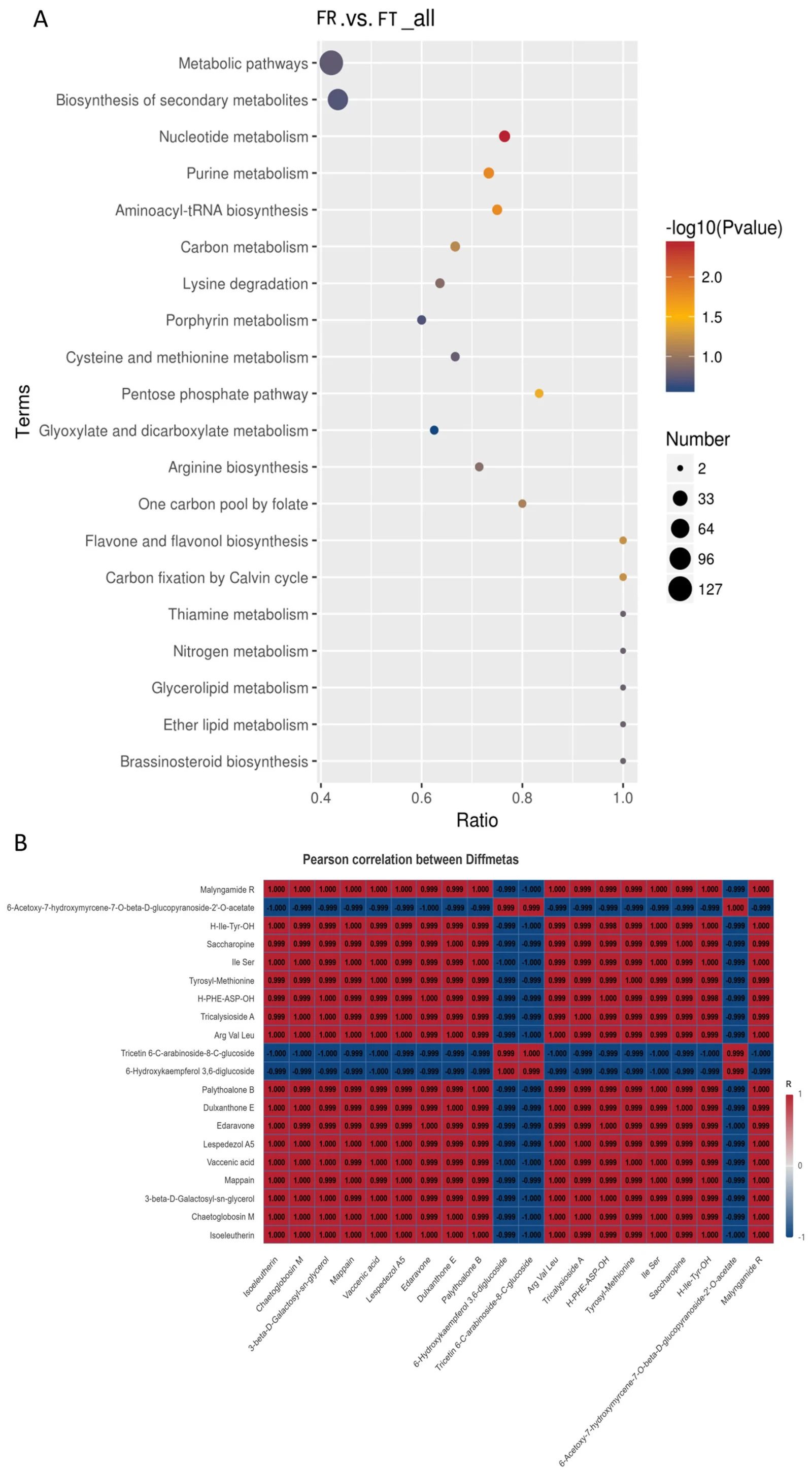
(**A**) KEGG pathway enrichment analysis of differential metabolites. (**B**) Correlation clustering heatmap of differential metabolites.

Findings from pathway enrichment analysis showed that nucleotide metabolism was the most significantly enriched pathway, followed by purine metabolism and aminoacyl-tRNA biosynthesis, suggesting these pathways as core metabolic hubs responding to treatments (Figure 6A). Carbon fixation in the Calvin cycle, flavone and flavonol biosynthesis, and phenylpropanoid biosynthesis were also highly enriched. These results collectively indicate that flower removal mainly affects nucleotide metabolism, amino acid metabolism, lipid metabolism, and secondary metabolism. When floral organ development demands substantial carbon and nitrogen resources for nucleotide synthesis (cell division) and aminoacyl-tRNA biosynthesis (protein synthesis), the enhancement of primary metabolism often suppresses secondary metabolism. For instance, it is hypothesized that carbon flux may be reallocated within the phenylpropanoid pathway to prioritize the energy and structural requirements of reproductive growth, which could consequently lead to a reduction or alteration in the accumulation of secondary metabolites such as flavonoids [25].

Flavone and flavonol biosynthesis exhibited the highest enrichment ratio, and number of differential metabolites across all pathways, indicating it as the core regulatory pathway responding to FT treatment. Other prominent pathways included carbon metabolism, amino acid biosynthesis, biosynthesis of secondary metabolites, and nitrogen metabolism, which corresponded well with the previously obtained data of quality indicators and amino acid quantification.

The correlation clustering heatmap reveals two significantly negatively correlated metabolite associations (Figure 6B). Flavonoid glycosides and flavonols exhibited an extremely strong positive correlation. In contrast, flavonoids had a significant negative correlation with certain phenolic compounds. This result suggests a potential explanation for the antagonistic accumulation pattern that total flavonoids increased while total phenols decreased under FT treatment: the observed accumulation patterns suggest a shift in the relative abundance of phenolic and flavonoid compounds following flower removal.

## 4. Discussion

In this study, the regulatory effects of FR and FT on the metabolic profiles and quality formation in *D. officinale* leaves were systematically analyzed by quantifying physiological parameters and employing nontargeted metabolomics. Flower removal significantly may alter source–sink relationships, carbon–nitrogen partitioning, and secondary metabolic flux, leading to the systematic remodeling of the chemical composition and amino acid profile and the leaf metabolome.

### 4.1. Flower Removal Treatment Targetedly Regulates the Accumulation of Medicinally Active Components in D. officinale Leaves

This study verified that flower removal treatment significantly elevated the contents of polysaccharides and total flavonoids in *D*. *officinale* leaves, while reducing the accumulation of total phenols. As the core pharmacodynamic components of *D. officinale*, polysaccharides possess immunomodulatory and antioxidant activities. Meanwhile, flavonoids were markedly enriched under FT treatment. Combined with metabolomic data, multiple flavanol derivatives including gossypetin and morin were drastically upregulated. These results suggested that flower retention, as a strategy to manipulate source–sink relationships, could reshape photosynthetic carbon partitioning and might divert more photosynthates toward two medicinally valuable secondary metabolites, namely polysaccharides and flavonoids. A study by Pan et al. suggested that the higher content of flavonoids in unflowering plants may be attributed to their massive consumption during flowering [26].

Correlation analysis revealed an extremely strong negative correlation between flavonoids and most simple phenolic compounds. FT treatment activated the key branch of flavonoid biosynthesis, may redirect more precursor phenylalanine toward flavonoid production and consequently lowering the content of free total phenols. It should be noted that while our untargeted metabolomics data reveals significant metabolic shifts, the precise physiological mechanisms underlying these changes remain hypothetical and warrant further investigation using targeted physiological and molecular approaches.

### 4.2. Flower Removal Profoundly Affects the Leaf Metabolome of D. officinale

Findings from PCA revealed a clear separation between the FR and FT groups, with approximately 2000 differential metabolites, indicating that flower removal could strongly regulate leaf metabolism. As a strong sink organ, the flower continuously competes for photosynthates, nitrogen, and carbon skeletons during flowering [27,28]. When the flowers were retained, more photosynthates were translocated to the flowers, and primary metabolism dominated the leaves. After flower removal, photosynthates and phenylpropanoid precursors accumulated in the leaves, and secondary metabolic pathways were significantly activated. These findings were highly consistent with our metabolomic results [29,30,31]. The increase in total flavonoids and polysaccharides in the FT group, along with opposite changes in the total phenols levels, is consistent with the enrichment of the phenylpropanoid, flavone, and flavonol biosynthesis pathways.

### 4.3. Flower Removal Improves the Medicinal Quality of Leaves by Enhancing Flavonoid Biosynthesis

Several flavonoid glycosides, quercetin derivatives, and schaftoside were significantly upregulated in the FT group, consistent with the increase in total flavonoids [31,32]. Flavonoids are the primary active components responsible for the antioxidant, anti-inflammatory, hepatoprotective, and immunomodulatory activities of *D. officinale* [33]. Upregulation of these metabolites in the FT group represents the core metabolic signature of this study. As *D. officinale* leaves are rich in flavonoids and have high biomass, they exhibit great development potential. Our findings indicated that flower removal further increased the flavonoid content and improved the medicinal quality of leaves, thereby establishing a scientific basis for the utilization of leaves.

The amino acid profile of flower-retaining (FR) plants is characterized by the enrichment of transport-type and primary metabolic amino acids, which sustains the high nitrogen demand required for flowering; by contrast, flower-removing (FT) plants are featured by the accumulation of storage-type amino acids and secondary metabolic precursor-type amino acids, which aligns with the metabolic traits of the vegetative growth stage. Interestingly, a similar phenomenon was observed in *Rosa chinensis.* Chen et al. demonstrated that the total content of 16 amino acids in rose exhibited an overall downward trend from the bud stage to the full-bloom stage, likely because nutrients were exported outward to promote the growth and anthesis of other flower buds. Although these are distinct species, this finding from another plant provides supporting evidence suggesting that the amino acid dynamics in *D. officinale* may also be associated with similar source–sink nutrient allocation mechanisms [34].

During traditional cultivation, the leaves of *D. officinale* are usually discarded despite their large biomass. Our findings revealed that flower removal significantly increased the levels of flavonoids in leaves, converting leaves from agricultural byproducts into high-value medicinal materials. Therefore, flower removal can be used as a precise regulation strategy in leaf-oriented cultivation systems to improve the development and enhancing the economic benefits of *D. officinale* leaves.

### 4.4. Nucleotide and Amino Acid Metabolism Are Core Regulatory Nodes for Flavonoid Accumulation

Findings from KEGG enrichment revealed purine metabolism, nucleotide metabolism, and aminoacyl-tRNA biosynthesis as the most significant core pathways. Nucleotide metabolism provides the basis for energy metabolism (adenosine triphosphate/adenosine diphosphate) and nucleic acid synthesis, whereas aromatic amino acids (Phe, Tyr) serve as direct precursors for flavonoid biosynthesis [35,36]. Enhanced secondary metabolism after flower removal increases the demand for nucleotides and amino acids, thereby strongly activating related pathways.

Quantitative amino acid analysis combined with metabolomic pathway results collectively suggested that flower retention triggered global reprogramming of nitrogen metabolism in leaves. Amino acids including arginine, lysine, histidine and tyrosine were significantly upregulated in the FT group. Arginine acts as the precursor of nitric oxide (NO) signaling, mediating plant stress responses and the activation of secondary metabolism; lysine and tyrosine supply nitrogen skeletons for the biosynthesis of flavonoids, laying a material foundation for massive flavonoid accumulation.

Although glutamic acid and GABA are well-documented stress-resistant bioactive amino acids, no significant intergroup differences were detected for them. Their homeostatic levels maintain basic osmotic balance in leaf cells and they are not involved in the FT-specific regulation of secondary metabolism. The above alterations in nitrogen metabolism serve as a vital intermediate link through which flower regulates secondary metabolic pathways.

## 5. Conclusions

The regulatory effects of flower retention on the quality and metabolome in *D. officinale* leaves were systematically explored in this study. Flower retention significantly altered the key quality indicators of *D. officinale* leaves with distinct regulatory patterns. Flower removal significantly increased the total flavonoid and amino acid content, including Tyr, Hylys, Lys, His, Arg, whereas flower retention promoted the accumulation of total phenols.

Flower removal dramatically remodeled the leaf metabolome, with 1999 significantly differential metabolites accounting for 39.5% of the total metabolites that were annotated. The FT group was characterized by an increase in the levels of flavonoids.

Nucleotide metabolism, purine metabolism, aminoacyl-tRNA biosynthesis, and flavonoid biosynthesis are the most statistically significant that respond to flower regulation. Collectively, these changes reveal the potential mechanisms underlying flower-mediated alterations in the leaf metabolic profile. However, it is important to note that this study only observed metabolite accumulation and did not directly confirm enhanced antioxidant or medicinal properties through pharmacological evaluation or bioactivity assays.

As a strong sink organ, the flower triggers a resource competition in leaves by regulating source–sink dynamics and carbon–nitrogen partitioning. Flower removal reduces the synthesis of structural lipids and suggests carbon skeletons to flavonoid biosynthesis, thereby altering the accumulation patterns of compounds associated with medicinal quality. Collectively, our findings provide a crucial theoretical reference and serve as a technical guide for the optimization of flower-thinning management in *D. officinale*, as well as the potential application value of its leaf biomass resources. It should be noted that the metabolomic analysis in this study was performed on leaves subjected to 80 °C drying treatment, rather than on fresh leaves. Therefore, some of the conclusions drawn herein may require further validation in future studies. Future studies involving direct bioactivity validation are warranted to establish the link between these metabolic changes and actual medicinal efficacy.

## Data Availability

The original contributions presented in this study are included in the article/Supplementary Materials. Further inquiries can be directed to the corresponding author.

## Supplementary Materials

The following supporting information can be downloaded at: https://www.mdpi.com/article/10.3390/metabo16090643/s1, Table S1. FR.vs.FT__all_with_BHP-1; Table S2. FR.vs.FT__all_with_BHP-2.
